# Comparison of Immune Response in Mice Immunized with Recombinant PreS2/S-C18-27 Protein Derived from Hepatitis B Virus with Commercial Vaccine

**DOI:** 10.30699/IJP.2022.553785.2896

**Published:** 2022-09-02

**Authors:** Elaheh Gholami Parizad, Abbas Ali Imani Fooladi, Hamid Sedighian, Elham Behzadi, Azar Valizadeh, Afra Khosravi

**Affiliations:** 1 *Clinical Microbiology Research Center, Ilam University of Medical Sciences, Ilam, Iran *; 2 *Applied Microbiology Research Center, Systems Biology and Poisonings Institute, Baqiyatallah University of Medical Sciences, Tehran, Iran *; 3 *Academy of Medical Sciences of the I.R. of Iran, Tehran, Iran*

**Keywords:** CpG 7909, Hepatitis B Core Antigen, Hepatitis B Vaccines, Hepatitis B Virus, preS2HBsAg

## Abstract

**Background & Objective:**

The vaccine available to prevent Hepatitis B virus disease is ineffective in 5% of people due to the use of HBsAg as a weak immunogenic factor. In the present study, PreS2/S fused to C18-27 peptide fragment as an effective antigen and is proposed as a promising vaccine candidate compared with the conventional vaccine prescribed in the vaccination program.

**Methods:**

After the synthesis of PreS2/S genes and C18-27 peptide fragment in pET28a, the recombinant protein was confirmed by Western blotting. The efficacy of the PreS2/S-C18-27 protein was compared with the conventional vaccine injected into five groups of rats. Finally, the cytokine level of IF-r, IL-2, IL-4, IL-10, TNF-a, IgG1, and IgG2a were measured using the ELISA method.

**Results:**

This study showed no significant difference between the recombinant vaccine group and PBS control group in the IF-r test, but there was a significant difference between groups testing IL-2 and IL-10. In addition, the group receiving the recombinant vaccine with CPG adjuvant at a dilution of 1/10 in the IgG total test on days 14 and 45 after the first injection showed a significant difference in comparison with other groups.

**Conclusion:**

This study showed no statistically significant difference between the recombinant protein vaccine group and the conventional vaccine group. The Th1- mediated immune responses obtained from recombinant proteins with and without CPG performed better than conventional vaccines, possibly due to the functional deficiency of the available vaccines.

## Introduction

Hepatitis B virus (HBV) is the smallest DNA virus known (1, 2). This small virus has so far been responsible for infecting more than two billion people throughout the world (2), whose virulence is 100 times greater than that of human immunodeficiency viruses (HIV) (3, 4).

Since the 1980s, when the hepatitis B virus surface antigen (HBsAg) was employed in developing recombinant vaccines, this fragment has been the basis of vaccination against the virus (5). The HBsAg and core protein of the virus are potential targets for producing and developing a hepatitis B vaccine (6, 7). HBsAg is composed of three glycosylated proteins: S (small), M (middle), and L (large) HBs. The L protein consists of three domains: s, preS1, and preS2. The M protein contains two domains called preS2 and s. The S protein also consists of only one domain. The pre-S domain (preS_1_, preS_2_) has antigenic epitopes based on which diagnostic and therapeutic systems have been developed (6). Despite a global cohesive vaccination program over the past few decades, the recombinant hepatitis B vaccine that is currently in use, remains low or with no response in some people. The degree of immunogenicity in individuals depends on factors such as age, sex, body mass index (BMI), smoking, underlying diseases such as diabetes, etc. (5). In addition, 5% of children who are vaccinated with the conventional vaccine may carry HBV in the future, the cause of which is still unknown (5).

In this regard, one of the solutions is to exploit different domains and fragments of surface antigen along with other fragments, including the core protein of the virus, and adding various adjuvants to the construction of a recombinant vaccine that can fill the gaps in the existing vaccine (5, 6, 8, 9).

Many studies have focused on improving vaccine immunogenicity by augmenting preS1and preS2 or HBcAg-containing nucleocapsids to the S protein to enhance vaccine efficacy.

Zuckerman* et al.* (1997) studied a recombinant vaccine containing preS1and preS2 in healthy subjects who responded well to the vaccine after receiving the routine three doses. Eventually, improvements in immunogenic factors in response to the recombinant vaccine became apparent (10).

Nowadays, efforts to find adjuvants with higher potency and quality in the antibody production industry have led to the identification of different types of adjuvants since the use of alum adjuvant as a dominant adjuvant in the formulation of most vaccines, including the hepatitis B vaccine. In most cases, alum adjuvant activates TH2 cell-mediated immune response that cannot completely clear virus-infected cells (11-14). In the same way, with the recognition of Toll-like receptors (TLRs) as bridges between innate and adaptive immune systems against microbes, the use of their agonists has been considered in the production of gene and recombinant vaccines. Cytosine-phosphate-guanine (CpG) is one of the most popular TLR agonists, whose fusion in the formulation of new vaccines is of great interest (9, 15, 16).

One of the TLR9 agonists is CpG 7909, which can directly activate B cells and pDCS and helps induce both innate and adaptive immune responses. In addition, the CpG is able to induce the secretion of potent TH1-derived cytokines such as interferon-gamma (IFN-γ) and, by secreting smaller amounts of TH2-derived cytokines, provide more T helper cells to both humoral and cell-mediated immune responses (9, 15-17). This increases the likelihood of viral clearance in people who may not respond well to vaccination.

Moreover, research has shown that Hepatitis B core antigen (HBcAg) can act on and activate B lymphocytes as primary antigen-presenting cells (APCs), and when combined with HBsAg, they have a synergistic effect on antibody production as well as cell-mediated responses. Among these, amino acids 18 to 27 of the hepatitis B virus core protein are known to be one of the most effective components for stimulating humoral and cell-mediated immunity, which has the ability to switch cell-mediated immunity to humoral immunity in accordance with numerous studies (13, 15, 18-20).

This study also investigated the use of preS2/S fragment along with c18-27 antigen peptide fragment of hepatitis B virus as a candidate for hepatitis B vaccine and co-injection of this recombinant protein with CpG7909 adjuvant and comparison of its immunogenicity with conventional HBV vaccine to improve the quality of conventional vaccines in Iran.

## Material and Methods

**Designing PreS2/S-C**_18-27_ **Construct and Plasmid**

**Fig. 1:**
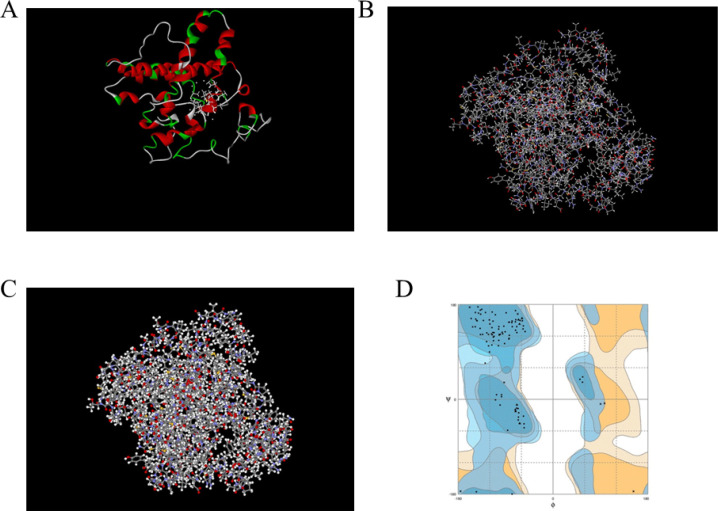
**A: **Solid ribbon views of the modeled protein. **B**: stick view of the modeled protein. (*yellow*= sulfur; *blue*= nitrogen; *gray*= carbon; *red*= oxygen **C:** solvent surface **D:**Ramachandran plot of the modeled protein. Number of residues in favored region (~ 98.0% expected): 168(56.6%). Number of residues in the allowed region (~2.0% expected):65(21.9%)

Sequences of preS2/S and C_18-27 _genes were extracted from GenBank (GenBank Accession No. CCH63722.1 and NO. AF324125.1, respectively), and different models were designed for the fusion of two genes with and without flexible linkers. Three-dimensional (3D) model structures were predicted using an online I-TASSER server (Figure1A-D), and the prediction of their secondary structure in terms of checking the coil or helix nature of structures conducted by online 2D-Structure servers. Ultimately, GC% and their optimizer were evaluated using J-CAT software (to express in *E. coli* as host) (21).

**In silico** **Modeling of PreS2/S-C**_18-27_** Protein**

Bioinformatics is a computational method used to design, discover, and develop new drugs and vaccines based on antibodies, aptamer, Immuno-toxins, and proteins (22-31). In this study, we used some *in silico* methods to validate and evaluate our designed proteins. Protein sequences were examined by CLC viewer 7.0 software and the online Expasy ProtParam server for factors such as hydrophobicity, solubility, environmental stability, and molecular weight. In addition, the overall antigenicity of the designed protein was investigated on the online VaxiJen server, which showed a number greater than 0.4 (Figure 1A-D).

**Fig. 2:**
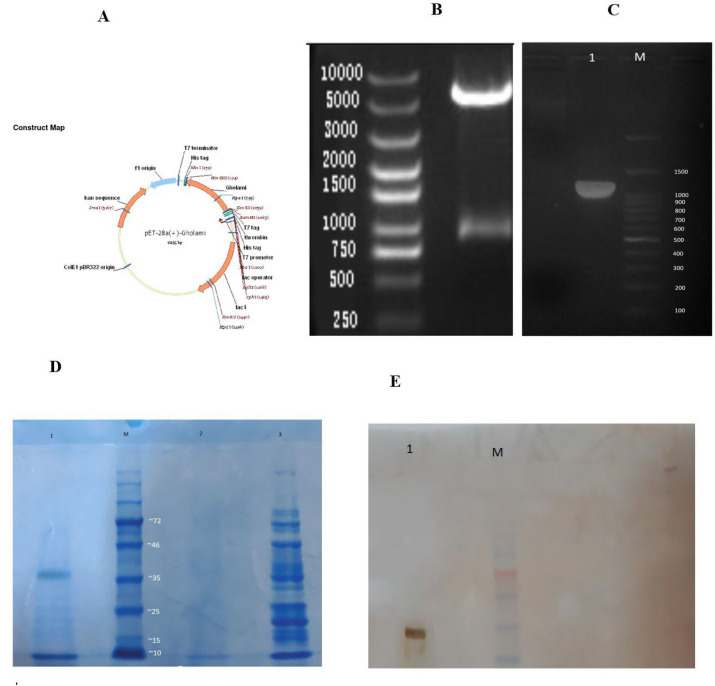
**A: **Construction of vector map (Gholami gen: PreS2/S-C18-27). **B**: Restriction digestion *Eco*RI/*Hin*dIII. **C:** Confirmation of transformed clones of *E. coli* by colony PCR with the 5^' ^T7 primer paired with 3' clone. LAN 1: PreS_2_/S-C_18-27 _was cloned in pET28a with 1191bp length (891 bp PreS2/S-C18-27 + 300bp pET28a vector). LAN M: DNA size marker (100bp).** D:** Purification and expression of the recombinant PreS2/2-C_18-27_ fusion protein. Analysis of the recombinant PreS2/S-C_18-27_ protein by SDS-PAGE, 12.5% stained with Coomassie blue. Lan 1: purification of the eluted recombinant protein of approximately 37 KDa. Lan M: protein marker(1KD). Lan 2: uninduced production. Lan 3: before extraction of product. **E: **Confirmation of purification PreS2/S-_C18-27 _by western blot analysis. Lan 1: protein extraction. Lan M: protein marker (1 KDa)

After reviewing, the 3D model of the linker-free structure was selected and designed in the expression vector of pET28a (Figure 2A), and the restriction enzymes that had a cleavage site in this gene structure were deleted using the Web cutter and Neb cutter online servers and re-checked in CLC software (Figure 2B). The N-terminal end of the structure was the site for the Eco RI enzyme, and the C-terminal end was the site for the Hind III enzyme. For easier purification, higher solubility, and better post-translational protein efficiency after cloning and expression processes, a His tag was added to the C-terminal end (5/EcoRI-PreS2/S-C_18-27_-HindIII). Figure 1 is a schematic of the fusion protein PreS2/S-C_18-27_: His and a map of the vector structure (30, 32-34)(Figure 2A).

**Gene Construction and Cloning**

The gene sequence designed for synthesis was sent to Biomatik (Canada). Gene subcloning in Pet28a, as an expression vector, was performed by the company itself. After receiving the subcloned and His tagged gene, multiple cloning sites in the pET28a vector were amplified using PCR and universal primers (Forward: 5′-CGAGCCCGATCTTCCCCATC-3′ and Reverse: 5′- GCTAGTTATTGCTCAGCGG-3′).

The thermal program of this process includes the initial denaturation of 94°C for 5 minutes and 25 cycles, including 94°C for 1 minute, 55°C for 45 seconds, 72°C for 1 minute, and finally, one cycle of 72°C for 5 minutes.

The obtained PCR product was resubmitted to Biomatik (Canada) for sequencing. In order to clone the gene construct, *E. coli* strain BL21 was treated with calcium chloride and CaCl_2_. The resulting colonies were harvested for PCR with ex-thermal conditions and universal primers of Pet28a.

After confirmation of the transformed cells, the plasmid was extracted using an RNA extraction kit (*iNtRON*, South Korea) according to kit instructions and electrophoresed on 1% agarose gel to re-confirm the transformation.

**Protein Expression Optimization and Production**

Bacteria containing the recombinant preS2/S-C_18-27_-Pet28a plasmid (with a concentration of 30 µg/mL) were inoculated in an LB broth medium containing 1.5% kanamycin and incubated for 16 hours in a shaker incubator at 37°C. This 18-hour culture was used for inoculation in fresh LB broth medium containing kanamycin. The medium was incubated in a shaker incubator, and when the turbidity of the medium reached 0.6, the expression of recombinant protein was induced with Isopropyl β- d-1-thiogalactopyranoside (IPTG) at different concentrations of 0.5 to 1 mM in 6 separate media. In addition, bacterial samples were taken from the induced media at 0, 2, 4, 6, and 8 hours. The expression level of the protein was evaluated at the mentioned times by electrophoresis on 12.5% polyacrylamide gel and then stained with Coomassie Brilliant Blue G-250 (Merck, Germany).

To produce the recombinant preS2/S-C_18-27_-Pet28a protein as an optimizer, a 16-hour culture of *E. coli* BL21 was prepared according to previously described and used for inoculation in 1 liter of fresh LB medium, then, the bacteria reached proper turbidity.

**Protein Purification**

After the time required for protein expression in the host bacterium, the bacterial cells were collected by centrifugation at 10000 rpm, and the pellet from the precipitate was washed with phosphate-buffered saline (PBS). Bacterial cell precipitate in buffer B (8 mM urea + 10 mM Tris-Cl + 100 mM NaH_2_PO_4_) was dissolved at pH 8.5 and centrifuged at 12,000 rpm for 30 minutes to separate the soluble phase from insoluble cell bodies. Then, nickel resin (Ni-NTA) (QIAGEN, Germany) was added to the solution phase and mixed for 2 hours. After transfer to a polystyrene column, the mixture was washed with buffer C (8 M urea + Tris-Cl + 100 mM NaH_2_PO_4_) at PH 6.3. Urea removal was performed using buffers containing urea with decreasing gradients (8, 6, 4, 2, 1, and 0 mol), and finally, the proteins were separated and collected from the column using 250 mM imidazole solution.

The protein solution was dialyzed versus PBS (pH 7.4) to remove imidazole for 36 hours. The amount of pure protein obtained was determined by spectrophotometry, and the total amount of soluble protein purified from one liter of the bacterial medium was calculated.

**SDS-PAGE and Western Blot Analysis**

The recombinant protein was transferred from polyacrylamide gel to polyvinylidene difluoride (PVDF) paper under semi-dry conditions (Bio-Rad, USA). Ponceau S-stained membrane was cut into thin strips to control and transfer the protein. The sealing of the PVDF membrane with Skim milk 3% solution was performed overnight. Then, the paper was washed with PBST, and a membrane was added to the papers with Anti His tag (SigmA A705, USA) at a dilution of 2000:1 and placed on a shaker for 45 minutes. Next, it was rinsed again with PBST buffer. The membrane was added to the papers with a solution of diaminobenzidine dye (DAB, SigmA, USA), given 15 minutes for the protein bands to appear, and the membrane was scanned (10).

**Subunit Vaccine: Preparation and Immunogenicity**

After desalination with Vivaspin20 columns (Sartorius Stedim Biotech, Germany) as well as LPS FREE using a kit (Qiagen, Switzerland), PreS2/S-C_18-27_ fusion protein was dissolved in PBS (0.2 mg/mL).

The female Balb/C mice aged 6 to 8 weeks were supplied from Baqiyatallah Research Center (Tehran, Iran), and the conventional recombinant HBV vaccine was obtained from Pasteur Institute in Tehran (Tehran, Iran). The CpG 7909 adjuvant was obtained from InvivoGen Company (USA).

The subcutaneous injection was performed in mice with a final volume of 1 mL. The animals were assigned to 5 groups of 7 (Table 1).

**ELISA for IFN-γ, IL-2,** **IL-4, IL-10, TNF-α, IgG 1 and IgG2a**

The mice were sacrificed two weeks after the final immunization (45 days after the first injection). The spleens of vaccinated and control mice were dissected under aseptic conditions and homogenized by a cell strainer. A cell suspension was prepared, and erythrocytes were removed by a lysis buffer (ammonium chloride). Extracted spleen cells were poured into 12-well plates at a density of 5×10^6^ cells per well cultured in 500 µL of RPMI1-640 medium, penicillin/streptomycin (Penstrep) solution (Biosera, UK), and 10% fetal bovine serum (Gibco, UK). The spleen cells in the microplates were divided into two treatment groups: (i) 10 µL of PreS2/S-C_18-27_ protein and (ii) 5 µg/mL of PHA. After 72 hours of incubation at 37°C, the supernatant of the culture medium was collected. The levels of IFN-γ, IL-2, IL-4, IL-10, and TNF-α cytokines, as well as IgG1 and IgG2a in the culture supernatant, were measured by ELISA kit (eBioscience, Austria) according to the manufacturer's instructions.

**Statistical Analysis**

Results are presented in mean ± SEM. Data were compared by one-way ANOVA using SPSS 16 (SPSS Inc., Chicago, IL., USA) at a statistically significant P-value≤0.05. Graphs were drawn using GraphPad Prism6 software. Statistical tests were repeated three times for each group. 

## Results

**Molecular Modeling of PreS2/S-C**_18-27_ **Recombinant Protein**

Initially, more than 20 models were obtained by online modeling software, and finally, one of them with the best ERRAT score was selected. Several models were developed at different stages of protein modeling analysis. During the modeling process, the proteins formed dimers. The solvent surface and solid ribbon were the structure of the final model (Figure 1A-D).

In Figures 2A, 2C, the final model had an ERRAT score of 97%. The Ramachandran diagram confirmed an acceptable structure with a validity of more than 98% (Figure 2D).

**Construction of recombinant fusion plasmids**

During the codon-optimization process, a pET-28a expression vector was placed at the C-terminal end (Figure 2A). EcoRI and HindIII enzyme cleavage sites as well as EcoRI/XhoI cloning sites, were used (Figure 2B). The presence of the PreS2/S-C_18-27 _gene fragment inside the recombinant plasmid was confirmed by colony PCR for about 20 colonies. The gene size, including the expression vector, was 1191 bp, and without the expression vector was 891 bp (Figure 2C). 

**Optimization of Recombinant Protein Production**

In order to find the optimal environmental conditions to achieve the highest protein production efficiency, the designed preS2-S/C_18-27_clones were subjected to different concentrations of IPTG, temperature ranges and incubation periods. Finally, SDS-PAGE analysis showed that a concentration of 0.9 M of IPTG at 37°C overnight is the optimal condition for achieving the highest efficiency of recombinant protein production (Figure 2D).

**Purification and Identification of Recombinant Fusion Protein**

Ni-NTA columns and His-tag linked to the recombinant protein were used to purify the protein. SDA-PAGE analysis confirmed that the purified recombinant preS2-_2_/C_18-27_protein has a band with a molecular weight of 37KDa (Figure 2D). This protein was then analyzed by the western blot method to determine that the preS2-_2_/C_18-27_ fusion protein has the ability to identify His-probe Antibody HRP Antibodies (Figure 2E).

**Cytokines** **assay in Spleen Cell Culture**

Cytokines IL-2, IL-4, IL-10, IFN-γ, and TNF-α were measured in a culture medium containing supernatant of each immunized group (7 mice in each group).

**IFN-γ Response**

**Fig. 3:**
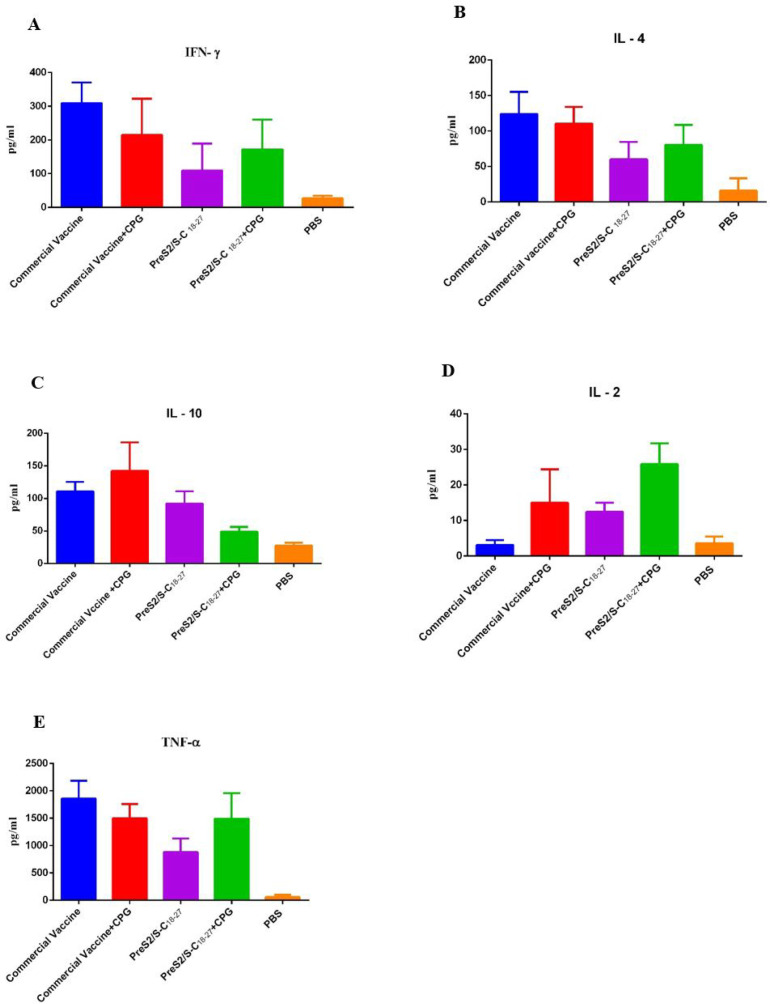
**A: **Cytokine release from splenocytes of immunized mice. IFN-γ secretion by splenocytes was evaluated following stimulation with PreS2/S-C_18-27_ and PHA. PBS and commercial vaccines used as control. Freshly isolated spleen cells were plated in duplicate at 5× 10^6^ cell per well in 24-well plate and incubated with PreS2/S-C_18-27_(10 µg/mL) and PHA(5 µg/mL) for 24 h at 37℃, 5% CO_2_. IFN-γ, TNF-a, IL-4, IL-10 and IL-2 were detected using a mouse, ELISA Kit. There is no significant difference between the groups. **B: **Cytokine release from splenocytes of immunized mice. IL-4 secretion by splenocytes was evaluated following stimulation with PreS2/S-C_18-27_ and PHA. PBS and commercial vaccines used as control. **C:** Cytokine release from splenocytes of immunized mice. IL-10 secretion by splenocytes was evaluated following stimulation with PreS2/S-C_18-27_ and PHA. PBS and commercial vaccines used as control. **D: **Cytokine release from splenocytes of immunized mice. IL-2 secretion by splenocytes was evaluated following stimulation with PreS2/S-C_18-27_ and PHA. PBS and commercial vaccines used as control. **E: **Cytokine release from splenocytes of immunized mice. TNF-a secretion by splenocytes was evaluated following stimulation with PreS2/S-C_18-27_ and PHA. PBS and commercial vaccines used as control

The splenic lymphocyte activity of mice immunized with preS2-S/C_18-27_ recombinant protein without adjuvant in vitro showed no significant difference after stimulation with the same protein compared with other groups. In addition, the difference was not significant in other groups, even between the group receiving the conventional vaccine with and without CpG adjuvant and the control group. However, the highest level belonged to the conventional vaccine group without CpG adjuvant (Figure 3A).

**IL-4 Response**

The highest level was found in the conventional vaccine group without CpG adjuvant. There was a significant difference between the group receiving PreS2-S/C_18-27_ without CpG adjuvant and the groups receiving a conventional vaccine with and without CpG (*P*<0.000, *P*=0.000, respectively).

In addition, the group receiving PreS2/S-C_18-27_ with CpG adjuvant had the lowest values among the experimental groups and showed a significant difference in comparison with the group receiving conventional vaccine without adjuvant (*P*=0.006) (Figure 3B).

Moreover, the groups receiving a conventional vaccine with and without CpG and those receiving PreS2-S/C18-27 protein with and without adjuvant were significantly different from the PBS group (*P*<0.000, *P*<0.000, *P*=0.000, *P*=0.017, respectively).

**IL-10 Response**

There was a significant difference in this regard between the group receiving a conventional vaccine with CpG adjuvant and the PBS group (*P*=0.024). 

In addition, there was also a significant difference between the group receiving a conventional vaccine with CpG adjuvant and the PreS2/S-C_18-27_ group, which had the lowest level of this cytokine (*P*=0.019) (Figure 3C).

**IL-2 Response**

There was a significant difference between the group receiving the PreS2/S-C_18-27_ recombinant protein with adjuvant and the group receiving conventional vaccine alone (without adjuvant) (*P*=0.04). In fact, the difference between the two groups was the lowest and highest groups.

None of the groups showed a significantly different from the control group (Figure 3D).

**TNF-α Response**

There was a significant difference between the two groups receiving the PreS2/S-C_18-27_ recombinant protein with adjuvant and the group receiving the same protein without adjuvant (*P*=0.000).

In addition, there was a significant difference between the group receiving conventional vaccine without adjuvant and the group receiving PreS2/S-C_18-27_ recombinant protein without adjuvant (*P*=0.002).

All groups receiving the conventional vaccine and recombinant protein, with or without adjuvant, showed a statistically significant difference compared to PBS (Figure 3E).

**Humoral Immune Response and Isotyping**

**IGG1 Response**

**Fig. 4:**
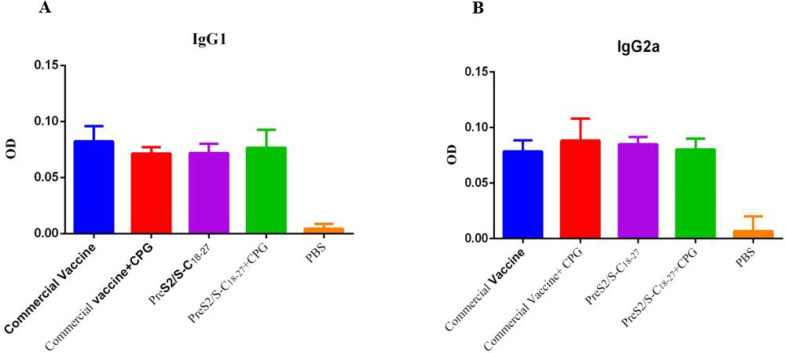
**A: **IgG1 release from splenocytes of immunized mice. **B:** IgG2a release by splenocytes of immunized mice

Differences were observed between the groups receiving PreS2/S-C_18-27_ with adjuvant and without adjuvant with the PBS control group (*P*<0.000). In addition, conventional vaccine groups with and without CpG were significantly different from the PBS group (*P*<0.000) (Figure 4A).

**IgG2a Response**

All groups receiving PreS2/S-C_18-27_ recombinant protein with and without adjuvant and conventional vaccine groups with and without adjuvant were significantly different from the PBS group (P<0.000) (Figure 4B).

**IgG Total in 14 Days after One Injection**

At a dilution of 1:10, a significant difference was observed between the conventional vaccine group without adjuvant and the group receiving PreS2/S-C_18-27_ recombinant protein with CpG adjuvant (*P*=0.012). In the same dilution, the group receiving PreS2/S-C_18-27_ recombinant protein with adjuvant showed a significant difference compared to the group receiving PreS2/S-C18-27 recombinant protein without adjuvant (*P*=0.004).

Furthermore, there was a significant difference (*P*<0.000) between the PreS2/2-C_18-27_ group with adjuvant, and the PBS group, while other groups receiving conventional vaccine showed no significant difference in comparison with the PBS group.

In the dilution 1:20, there was a significant difference between the recipients of PreS2/S-C_18-27_ recombinant protein with and without adjuvant (*P*=0.03).

**Fig. 5:**
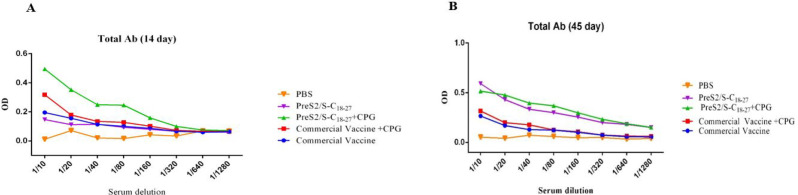
**A:** Total antibody 14 days after the first injection. PreS2/S-C18-27 with and without CPG groups compared with commercial vaccine groups. **B:** Total antibody 45 days after the first injection. PreS2/S-C18-27 with and without CPG groups compared with commercial vaccine groups

In other dilutions (1:40 to 1:1280), no significant difference was observed on this day (Figure 5A).

**Total IgG in 45 Days after One Injection**

On day 45, after initial injection (day zero) between the five experimental groups, only a significant difference was observed between the group receiving PreS2/S-C_18-27_ recombinant protein with adjuvant and PBS group at a dilution of 1:10 (*P*=0.047), and there was no significant difference between any of the eight dilutions (1:10 to 1:1280) (Figure 5B).

**Table 1:** Experimental Groups of the Study

Group Number	Injection Formula	Dose	Rout Of Injection
1	PreS2/S-C_18-27_	10 µg	subcutaneously
2	Commercial Vaccine	10 µg	subcutaneously
3	PreS2/S-C_18-27_ + 1 µg/mL CPG7909	10 µg	subcutaneously
4	Commercial Vaccine +1µg/mL CPG2709	10 µg	subcutaneously
5	PBS	-	-

## Discussion

According to the World Health Organization (WHO), more than 50 million people are diagnosed with acute hepatitis B infection yearly, and more than 240 million suffer from chronic hepatitis infection (6-12). 

Despite a comprehensive vaccination program in most developed and developing countries, hepatitis B infection is still a major problem, especially in Asia-Pacific regions (35, 36). The hepatitis B vaccine for adults has several limitations that cause no response or low response to the vaccine. About 10% of these unresponsive individuals are healthy individuals for whom no specific reason for their unresponsiveness has been established yet (37, 38). Perhaps one of the major problems of conventional vaccines is the use of genotype A, subtype A2, and serotype ayw2 for vaccine design, which is common in Northern Europe and the United States (36-40), whereas 99% of hepatitis B infections are caused by genotypes other than A2 and cross seroprotection occurs when the antibody titer is produced in the shortest possible time and with the maximum amount against the common antigens of each region, to prevent infection with other virus genotypes (32, 35, 39-41). Therefore, efforts are now being made to produce hepatitis B vaccine according to the common genotype and conditions in each country, to the extent that the vaccines in countries such as Korea and Russia are produced and consumed according to the common genotype in these countries (32, 35, 39-41). Hence, production and quality improvement of hepatitis B vaccine are one of the health priorities according to the common genotype in Iran, i.e., genotype D (42).

One of the components considered in recent years is preS2-S, known as M-protein. This protein consists of an mRNA with a molecular weight of 2.1 kbp, and the resulting protein will eventually contain 281 amino acids which play a significant role both in enhancing immunogenicity and in accelerating the secretion of antibodies against HBsAg due to the presence of the preS2 fragment (14, 33, 38, 43). In the structure of this protein 55, amino acids and an N-glycosylated site at its fourth amino acid position are found. These fragments have been evaluated alone or in combination with S protein in various studies and are currently used in third-generation vaccines and several research-based vaccines (33, 43, 44).

The S protein, which contains 226 amino acids, is important in the preparation of vaccines as well as diagnostic kits due to its "a" region from point 122 to 146 in this domain and has been conserved in different genotypes. Therefore, M protein with S and S_2_ fragments is a suitable target for developing new therapeutic and prophylactic vaccines by expanding and amplifying the specific responses of B and T cells (12, 32, 34, 39, 45, 46).

In addition, various studies have demonstrated that HBcAg can act on and activate B lymphocytes as APCs, while combining with HBsAg causes a synergistic effect on antibody production and cell-mediated responses (47). Among these, amino acids 18 to 27 of hepatitis B virus core protein have shown the greatest potency in stimulating humoral and cell-mediated immunity, which can switch cell-mediated immunity to humoral immunity (36).

Nowadays, scientists are trying to find adjuvants with higher potency and quality in antibody production, which has led to the identification of different types of new adjuvants (17, 48-50). In this direction, recognizing the function of TLRs as bridges between innate and acquired immunities against microbes has led to efforts to employ their agonists in developing recombinant and gene vaccines (33, 43, 44). During HBV infection, the TLR9 expression decrease, and since TLR9 is a very effective factor in the viral DNA presentation to dendritic cells, a reduction in its expression will result in an incomplete immune response to the virus and thus the progression of the disease to become acute will not be unexpected (43, 51, 52). The CpGs are nucleotide sequences that are considered agonists of TLRs, and the TLR9 agonist is CpG7909. This CpG directly activates B cells and pDCS and helps induce both innate and adaptive immune responses (48, 49). In addition, this CpG can induce the release of potent Th1-derived cytokines such as IL-12 and IFN-γ and, by secreting smaller amounts of Th2-derived cytokines, provide more T-helpers to both cell-mediated and humoral immune responses (43, 52). Thus, the likelihood of viral clearance from the body will also increase (34). Among the available agonists, CpG7909 has been approved by the USA Food and Drug Administration (FDA) and is now exploited as an adjuvant in vaccines such as the tetanus vaccine and even in some countries in the hepatitis B vaccine (48).

Since the conventional vaccine placed more emphasis on stimulating the immune response based on the stimulation of antibodies and Th1, and as this factor causes the vaccine to remain in asymptomatic carriers without an adequate immune response (50), this study aimed to reinforce cell-mediated response along with antibody responses to maintain cell-mediated responses while maintaining antibody responses that are desirable in the conventional vaccine, to include therapeutic application in addition to prophylactic use.

The IFN-γ is a cytokine secreted by Th1 cells, whose non-production has been observed in people who do not respond to the hepatitis vaccine or in people with this infection (12, 32, 35, 39, 45, 46).

In the present study, there were no significant differences in IFN-γ levels between the PreS2/S-C_18-27_ recombinant protein group, the conventional vaccine groups, and the PBS group. This significant difference was not observed between the conventional vaccine groups with or without CpG adjuvant and the PBS group (Figure 3A). Although these results confirm that the PreS2/S-C18-27 fusion protein does not stimulate the Th1 response more than other groups, it can be concluded that this recombinant protein did not act more powerfully than the conventional vaccine. In a study conducted by Li* et al.* (2015), the HBsAg fragments and HBcAg along with CpG were applied to develop a new therapeutic vaccine so that the mice receiving the above combination showed higher levels of IFN-γ than other groups receiving without CpG; however, these levels were not significant in this study (53).

Since IL-2 is also a cytokine affecting Th1 responses, an assay of this interleukin indicated a significant difference between the group receiving the PreS2/S-C_18-27_ recombinant protein formulated with CpG adjuvant and the group receiving conventional vaccine without CpG (*P*=0.04) (Figure 3D). As shown in Figure 3D, the level of this cytokine was not significantly different between the conventional vaccine group with and without CpG adjuvant and the PBS group. This may indicate that the PreS2/S-C_18-27_ recombinant protein with CpG adjuvant can more activate IL-2-stimulated Th1-based immune responses than other groups.

In a study done by Wang* et al.*, two weeks after the last dose of HBc_18-27__HIV Tat _49-57_ fusion vaccine, the serum levels of IFN-γ and IL-2 in the mice receiving this recombinant fusion with CpG were significantly higher than in the other groups. The reason for this difference can be found in different doses as well as differences in various fusion combinations (13) (Figure 3D).

New findings demonstrate that the best model for immune responses against viral infections is a combination of Th1 and Th2-based responses. The imbalance of Th1/Th2 responses induces the production of anti-inflammatory cytokines such as IL-4 and IL-10 (12, 39).

IL-10, which reduces the Th1 response and inhibits inflammatory and immune responses, demonstrated the difference between the two groups receiving PreS2/S-C_18-27_+CpG adjuvant and the conventional vaccine+CpG in this study. The highest production level of this cytokine belonged to the conventional vaccine+CpG group, and the lowest level belonged to the recombinant protein+CpG group (Figure 3C). As IL-10 often has an inhibitory and controlling role on the immune system, there was a significant difference in the production level of this cytokine when comparing the PBS group with the same conventional vaccine group+CpG. Next, the conventional vaccine group without adjuvant produced more IL-10, suggesting that the recombinant protein has shown better performance in producing inhibitory cytokines to promote immune responses rather than inhibiting them.

In addition, the production of IL-4, as an anti-inflammatory cytokine, promotes Th2-related reactions and differentiates and produces IgG. The measurement of this interleukin among the experimental groups revealed a significant difference between the groups receiving the PreS2/S-C_18-27_ recombinant protein±CpG and conventional vaccine±CpG. Since the conventional vaccine±CpG group showed a significant difference compared to the PBS control group, it can be inferred that the PreS2/S-C_18-27_ recombinant protein has a better balance for Th1/Th2 responses than the conventional vaccine (Figure 3B).

As IFN-γ production showed no significant difference (Figure 3A) and the IL-2, which acts in conjunction with IFN-γ (perhaps with less potency) due to the resulting recombinant fusion protein (Figure 3D), therefore more emphasis should be placed on this recombinant protein than the conventional vaccine. In addition, in terms of stimulation of Th2-based immune responses examined by IL-4 assay, our results showed better performance of the PreS2/S-C_18-27_ recombinant protein than conventional vaccine compared to the control group (Figure 3B).

Recent findings have proven that the best model for immune responses against viral infections is a combination of Th1- and Th2-based responses (12).

Tumor necrosis factor-alpha (TNF-α) is a proinflammatory, multivalent cytokine that plays an important role in developing a host immune response to HBV. One of the reasons for no or low response to the HBV vaccine is the polymorphism in the promoter of this cytokine, which can eventually lead to chronic hepatitis B infections. Studies have shown that cytotoxic T lymphocytes (CTLs) play a major role in overcoming the acute infection of the hepatitis B virus in hepatocytes. In this process, IFN-γ and TNF-α derived from CTLs and macrophages of the liver can inhibit the expression of viral genes and prevent viral replication in infected hepatocytes. However, high levels of this cytokine also cause tissue damage (39).

An increase in TNF-α level (seven months after vaccination) in healthy people vaccinated with conventional vaccines over time has been shown to be a positive functional indicator because the level of this cytokine decreases in HIV-positive people after vaccination, as one of the reasons for reducing the effect of the vaccine in these people (13). This study showed a significant difference between the conventional vaccine group without CpG and the PreS2/S-C_18-27_ recombinant protein group without CpG. There was even a significant difference between the PreS2/S-C_18-27_ group without adjuvant and with the same group - but without adjuvant - which was inconsistent with other studies in this field (13, 54) (Figure 3E).

The results of this study indicated that IgG2a, the production of which eventually leads to the activation of Th1 cells, in the experimental group receiving PreS2/S-C_18-27_ without adjuvants was significantly different from the control group (*P*=0.01), and this difference was not seen in other test and control groups. In this test, the highest level belonged to the conventional vaccine plus CpG group, and the lowest level was related to the group receiving vaccine alone, which in turn confirms that the current vaccine used alone does not have sufficient potency to produce IgG2a compared to the other groups tested (Figure 4B).

In a study by Davis* et al.* (17), the groups that also received CpG showed the greatest difference in IgG2a from the other groups.

Moreover, two weeks after the first dose of injection (14 days) in 1:10 and 1:20 dilutions (Figure 5A), there was a significant difference in the total IgG test between the two groups receiving the PreS2/S-C_18-27_ recombinant protein with or without adjuvant. The group receiving recombinant protein with adjuvant showed higher values in these two dilutions on day 14. A comparison of these two groups on day 45 after the first injection (two weeks after the second injection) (Figure 5B) did not show a significant difference. In addition, no difference was observed in this marker between other control and test groups.

## Conclusion

Although this study tested only one single dose and two injections for both conventional vaccines and produced recombinant protein, recombinant protein results showed a slight statistically difference with conventional HBV vaccine. However, the produced recombinant protein- with and without CpG adjuvant- clearly may perform better concerning Th1-based immune responses. By changing the dose, different formulations of the recombinant protein and adjuvant, or the number of injections, it may be possible to achieve a more effective outcome for the PreS2/S-C_18-27_ recombinant protein than for the conventional vaccine. In addition, Researches focusing on the effect of PreS2/S-C_18-27_ protein on transgenic mice with chronic HBV could be helpful to investigate this protein's potential efficacy in viral clearance.

## Conflict of Interest

The authors declared no conflict of interest.

## Compliance with Ethical Standards

The experimental protocol was reviewed, approved, and supervised by the institutional animal care and use committee of Ilam University of Medical Sciences (permit number:IR.MEDIL AM.REC.1395.105). During the experiments, the vaccinated mice were monitored every day. All surgery was performed under sodium pentobarbital anesthesia, and all efforts were made to minimize the suffering of animals. Mice were killed by cervical dislocation.

## References

[1] Nassal M (2008). Hepatitis B viruses: reverse transcription a different way. Virus Res.

[2] Locarnini S, Hatzakis A, Chen DS, Lok A (2015). Strategies to control hepatitis B: Public policy, epidemiology, vaccine and drugs. J Hepatol.

[3] Brechot C (2004). Pathogenesis of hepatitis B virus-related hepatocellular carcinoma: old and new paradigms. Gastroenterology.

[4] Tang B, Kruger WD, Chen G, Shen F, Lin WY, Mboup S (2004). Hepatitis B viremia is associated with increased risk of hepatocellular carcinoma in chronic carriers. J Med Virol.

[5] Lavanchy D (2005). Worldwide epidemiology of HBV infection, disease burden, and vaccine prevention. J Clin Virol.

[6] Azizi F, Janqurbani M, H H Epidemilogy and control common disease in Iran. Khosravi publication.

[7] Azizi M, Musacchio A, Pardo O, Figueroa N, Muzio V (2000). Expression of Hepatitis B Virus Core Antigen in Native and Fusion Forms in E coli. Iran Biomed J.

[8] Ni YH, Chang MH, Wu JF, Hsu HY, Chen HL, Chen DS (2012). Minimization of hepatitis B infection by a 25-year universal vaccination program. J Hepatol.

[9] Chen J, Liang Z, Lu F, Fang X, Liu S, Zeng Y (2011). Toll-like receptors and cytokines/cytokine receptors polymorphisms associate with non-response to hepatitis B vaccine. Vaccine.

[10] Tajiri K, Shimizu Y (2015). Unsolved problems and future perspectives of hepatitis B virus vaccination. World J Gastroenterol.

[11] Lobaina Y, Hardtke S, Wedemeyer H, Aguilar JC, Schlaphoff V (2015). In vitro stimulation with HBV therapeutic vaccine candidate Nasvac activates B and T cells from chronic hepatitis B patients and healthy donors. Mol Immunol.

[12] Jing M, Wang J, Zhu S, Ao F, Wang L, Han T (2016). Development of a more efficient hepatitis B virus vaccine by targeting hepatitis B virus preS to dendritic cells. Vaccine.

[13] Li J, Ge J, Ren S, Zhou T, Sun Y, Sun H (2015). Hepatitis B surface antigen (HBsAg) and core antigen (HBcAg) combine CpG oligodeox-ynucletides as a novel therapeutic vaccine for chronic hepatitis B infection. Vaccine.

[14] Seeger C, Mason WS (2015). Molecular biology of hepatitis B virus infection. Virology.

[15] Wang S, Han Q, Zhang G, Zhang N, Li Z, Chen J (2011). CpG oligodeoxynucleotide-adjuvanted fusion peptide derived from HBcAg epitope and HIV-Tat may elicit favorable immune response in PBMCs from patients with chronic HBV infection in the immunotolerant phase. Int Immunopharmacol.

[16] Bode C, Zhao G, Steinhagen F, Kinjo T, Klinman DM (2011). CpG DNA as a vaccine adjuvant. Expert Rev Vaccines.

[17] Cooper C, Davis H, Morris M, Efler S, Al Adhami M, Krieg A (2004). CPG 7909, an immunostimulatory TLR9 agonist oligodeoxy-nucleotide, as adjuvant to Engerix-B® HBV vaccine in healthy adults: A double-blind phase I/II study. J Clin Immunol.

[18] Chen X, Tang Y, Zhang Y, Zhuo M, Tang Z, Yu Y (2014). Tapasin modification on the intracellular epitope HBcAg 18-27 enhances HBV-specific CTL immune response and inhibits hepatitis B virus replication in vivo. Lab Invest.

[19] Wang S, Han Q, Zhang N, Chen J, Liu Z, Zhang G (2010). HBcAg18-27 epitope fused to HIV-Tat 49-57 adjuvanted with CpG ODN induces immunotherapeutic effects in transgenic mice. Immunol Lett.

[20] Shahrokhi N, Bouzari S, Jafari AAF (2006). Priming Hepatitis B Surface (Hbsag)- and Core Antigen (Hbcag)-Specific Immune Responses by Chimeric, Hbcag with A Hbsag 'A' Determinant. Iran Biomed J.

[21] Mahboobi M, Sedighian H, Malekara E, Khalili S, Rahbar MR, Zanoos KA (2021). Harnessing an Integrative In Silico Approach to Engage Highly Immunogenic Peptides in an Antigen Design Against Epsilon Toxin (ETX) of Clostridium perfringens. Int J Pept Res Ther.

[22] Mahboobi M, Sedighian H, Hedayati CH M, Bambai B, Esmaeil Soofian S, Amani J (2017). Applying bioinformatic tools for modeling and modifying type II E coli L-Asparginase to present a better therapeutic agent/drug for acute lymphoblastic leukemia. Intl J Cancer Manag.

[23] Mahboobi M, Sedighian H, Malekara E, Khalili S, Rahbar MR, Zanoos KA Harnessing an Integrative In Silico Approach to Engage Highly Immunogenic Peptides in an Antigen Design Against Epsilon Toxin (ETX) of Clostridium perfringens. Int J Pept Res Ther.

[24] Maleki F, Sadeghifard N, Sedighian H, Bakhtiyari S, Hosseini HM, Fooladi AAI (2020). TGFαL3-SEB fusion protein as an anticancer against ovarian cancer. Eur J Pharmacol.

[25] Moghadam ZM, Halabian R, Sedighian H, Behzadi E, Amani J, Fooladi AAI (2019). Designing and analyzing the structure of DT-STXB fusion protein as an anti-tumor agent: an in silico approach. Iran J Pathol.

[26] Mohseni Z, Sedighian H, Halabian R, Amani J, Behzadi E, Imani Fooladi AA (2021). Potent in vitro antitumor activity of B-subunit of Shiga toxin conjugated to the diphtheria toxin against breast cancer. Eur J Pharmacol.

[27] Jahangiri A, Amani J, Halabian R (2018). In silico analyses of staphylococcal enterotoxin B as a DNA vaccine for cancer therapy. Int J Pept Res Ther.

[28] Goleij Z, Mahmoodzadeh Hosseini H, Sedighian H, Behzadi E, Halabian R, Sorouri R (2020). Breast cancer targeted/ therapeutic with double and triple fusion Immunotoxins. J Steroid Biochem Mol Biol.

[29] Sedighian H, Halabian R, Amani J, Heiat M, Taheri RA, Imani Fooladi AA (2018). Manufacturing of a novel double-function ssDNA aptamer for sensitive diagnosis and efficient neutralization of SEA. Anal Biochem.

[30] Sedighian H, Halabian R, Amani J, Heiat M, Amin M, Fooladi AAI (2018). Staggered Target SELEX, a novel approach to isolate non-cross-reactive aptamer for detection of SEA by apta-qPCR. J Biotechnol.

[31] Hedayati Ch M, Amani J, Sedighian H, Amin M, Salimian J, Halabian R (2016). Isolation of a new ssDNA aptamer against staphylococcal enterotoxin B based on CNBr-activated sepharose-4B affinity chromatography. J Mol Recognit.

[32] Jeong GU, Ahn B-Y, Jung J, Kim H, Kim T-H, Kim W (2020). A recombinant human immunoglobulin with coherent avidity to hepatitis B virus surface antigens of various viral genotypes and clinical mutants. PloS one.

[33] Shen K, Shen L, Wang J, Jiang Z, Shen B (2015). Understanding Amino Acid Mutations in Hepatitis B Virus Proteins for Rational Design of Vaccines and Drugs. Adv Protein Chem Struct Biol.

[34] King TH, Kemmler CB, Guo Z, Mann D, Lu Y, Coeshott C (2014). A whole recombinant yeast-based therapeutic vaccine elicits HBV X, S and Core specific T cells in mice and activates human T cells recognizing epitopes linked to viral clearance. PloS one.

[35] Nishikawa K, Kimura K, Kanda Y, Sugiyama M, Kakihana K, Doki N (2020). A prospective trial of vaccine to prevent hepatitis B virus reactivation after hematopoietic stem cell transplantation. Bone Marrow Transplant.

[36] Walayat S, Ahmed Z, Martin D, Puli S, Cashman M, Dhillon S (2015). Recent advances in vaccination of non-responders to standard dose hepatitis B virus vaccine. World J Hepatol.

[37] Schmitt S, Glebe D, Alving K, Tolle TK, Linder M, Geyer H (1999). Analysis of the pre-S2 N- and O-linked glycans of the M surface protein from human hepatitis B virus. J Biol Chem.

[38] Toita R, Kawano T, Kang JH, Murata M (2015). Applications of human hepatitis B virus preS domain in bio- and nanotechnology. World J Gastroenterol.

[39] Backes S, Jager C, Dembek CJ, Kosinska AD, Bauer T, Stephan AS (2016). Protein-prime/modified vaccinia virus Ankara vector-boost vaccination overcomes tolerance in high-antigenemic HBV-transgenic mice. Vaccine.

[40] Kutinova L, Nemeckova S, Hamsikova E, Press M, Zavadova H, Hirsch I (1990). A recombinant vaccinia virus expressing hepatitis B virus middle surface protein Restricted expression of HBV antigens in human diploid cells. Arch Virol.

[41] Greiner VJ, Ronzon F, Larquet E, Desbat B, Esteves C, Bonvin J (2012). The structure of HBsAg particles is not modified upon their adsorption on aluminium hydroxide gel. Vaccine.

[42] Khodadad N, Seyedian SS, Moattari A, Biparva Haghighi S, Pirmoradi R, Abbasi S (2020). In silico functional and structural characterization of hepatitis B virus PreS/S-gene in Iranian patients infected with chronic hepatitis B virus genotype D. Heliyon.

[43] Schulze A, Schieck A, Ni Y, Mier W, Urban S (2010). Fine mapping of pre-S sequence requirements for hepatitis B virus large envelope protein-mediated receptor interaction. J Virol.

[44] Riu Garcia A (2007). Functional Analysis of Hepatitis B Virus Variants with Mutations in the Envelope Proteins: Staats-und Universitätsbibliothek Hamburg Carl von Ossietzky.

[45] Gholizadeh M, Khanahmad H, Memarnejadian A, Aghasadeghi MR, Roohvand F, Sadat SM (2015). Design and expression of fusion protein consists of HBsAg and polyepitope of HCV as an HCV potential vaccine. Adv Biomed Res.

[46] Inoue T, Tanaka Y (2020). Cross-Protection of Hepatitis B Vaccination among Different Genotypes. Vaccines.

[47] Gebbing M, Bergmann T, Schulz E, Ehrhardt A (2015). Gene therapeutic approaches to inhibit hepatitis B virus replication. World J Hepatol.

[48] McCluskie MJ, Davis HL (1998). Cutting edge: CpG DNA is a potent enhancer of systemic and mucosal immune responses against hepatitis B surface antigen with intranasal administration to mice. J Immunol.

[49] Eng NF, Bhardwaj N, Mulligan R, Diaz-Mitoma F (2013). The potential of 1018 ISS adjuvant in hepatitis B vaccines: HEPLISAV™ review. Hum Vaccines Immunother.

[50] Liang TJ, Block TM, McMahon BJ, Ghany MG, Urban S, Guo JT (2015). Present and future therapies of hepatitis B: From discovery to cure. Hepatology.

[51] Pollicino T, Cacciola I, Saffioti F, Raimondo G (2014). Hepatitis B virus PreS/S gene variants: pathobiology and clinical implications. J Hepatol.

[52] Julithe R, Abou-Jaoude G, Sureau C (2014). Modification of the hepatitis B virus envelope protein glycosylation pattern interferes with secretion of viral particles, infectivity, and susceptibility to neutralizing antibodies. J Virol.

[53] Schmitt S, Glebe D, Alving K, Tolle TK, Linder M, Geyer H (1999). Analysis of the Pre-S2 N- and O-Linked Glycans of the M Surface Protein from Human Hepatitis B Virus*. J Biol Chem.

[54] Gerlich WH (2015). Prophylactic vaccination against hepatitis B: achievements, challenges and perspectives. Med Microbiol Immunol.

